# Outcomes of a community-led online-based HIV self-testing demonstration among cisgender men who have sex with men and transgender women in the Philippines during the COVID-19 pandemic: a retrospective cohort study

**DOI:** 10.1186/s12889-022-12705-z

**Published:** 2022-02-21

**Authors:** Patrick C. Eustaquio, Roberto Figuracion, Kiyohiko Izumi, Mary Joy Morin, Kenneth Samaco, Sarah May Flores, Anne Brink, Mona Liza Diones

**Affiliations:** 1 LoveYourself, Inc, 715 Anglo Bldg., Shaw Blvd, 1550 Mandaluyong City, Philippines; 2 Family Planning Organization of the Philippines, Inc, Iloilo Chapter – Rajah Community Center, 2F Dulalia Building, Rizal St, 5000 Iloilo City, Iloilo City Proper Philippines; 3 World Health Organization, Philippines, Ground Floor Building 3 San Lazaro Compound, C. S. Gatmaitan Ave, Santa Cruz, 1000 Manila, Metro Manila Philippines; 4 Department of Health, Philippines, Compound San Lazaro St, Santa Cruz, 1000 Manila, Metro Manila Philippines; 5 Independent consultant, London, UK

**Keywords:** HIV self-testing, Cisgender men who have sex with men, Transgender women, Community-based interventions, Digital health, Differentiated service delivery, Philippines, Low- and middle-income countries

## Abstract

**Introduction:**

The Philippines, which has the fastest rising HIV epidemic globally, has limited options for HIV testing and its uptake remains low among cisgender men who have sex with men (cis-MSM) and transgender women (TGW), especially amid the COVID-19 pandemic. As HIV self-testing (HIVST) and technology-based approaches could synergize to expand uptake of HIV testing, we aimed to evaluate the outcomes of a community-led online-based HIVST demonstration and to explore factors associated with HIVST-related behaviours and outcomes.

**Methods:**

We did a secondary data analysis among cis-MSM and TGW who participated in the HIVST demonstration, who were recruited online and tested out-of-facility, in Western Visayas, Philippines, from March to November 2020. We reviewed data on demographics, sexuality-, and context-related variables. Using multivariable logistic regression, we tested for associations between the aforementioned covariates and two primary outcomes, opting for directly-assisted HIVST (DAH) and willingness to secondarily distribute kits.

**Results:**

HIVST kits were distributed to 647 individuals (590 cis-MSM, 57 TGW), 54.6% were first-time testers, 10.4% opted DAH, and 46.1% were willing to distribute to peers. Reporting rate was high (99.3%) with 7.6% reactivity rate. While linkage to prevention (100%) and care (85.7%) were high, pre-exposure prophylaxis (PrEP) (0.3%) and antiretroviral therapy (ART) (51.0%) initiation were limited. There were no reports of adverse events. Those who were employed, had recent anal intercourse, opted for DAH, not willing to secondarily distribute, and accessed HIVST during minimal to no quarantine restriction had significantly higher reactivity rates. Likelihood of opting for DAH was higher among those who had three or more partners in the past year (aOR = 2.01 [CI = 1.01–4.35]) and those who accessed during maximal quarantine restrictions (aOR = 4.25 [CI = 2.46–7.43]). Odds of willingness to share were higher among those in urban areas (aOR = 1.64 [CI = 1.15–2.36]) but lower among first-time testers (aOR = 0.45 [CI = 0.32–0.62]).

**Conclusions:**

HIVST could effectively reach hard-to-reach populations. While there was demand in accessing online-based unassisted approaches, DAH should still be offered. Uptake of PrEP and same-day ART should be upscaled by decentralizing these services to community-based organizations. Differentiated service delivery is key to respond to preferences and values of key populations amid the dynamic geographical and sociocultural contexts they are in.

## Introduction

The limited demand for HIV testing among the key populations (KP) has challenged the Philippines to reverse its HIV epidemic, where annual incidence of new infections and AIDS-related death increased by 237 and 315%, respectively, over the past decade [1]. Although estimated national prevalence is at 0.2%, the epidemic is concentrated among KP with prevalence disproportionately higher among people who inject drugs (PWID) (29.0%), cisgender men who have sex with men (cis-MSM) (5.0%), transgender women (TGW) (4.9%), and female sex workers (0.6%) [2]. Improvements in the first 95% of the UNAIDS 95–95-95 targets were noted in the recent years until the COVID-19 pandemic has decreased HIV tests done by 61% in 2020, ultimately leading to 68% of estimated people living with HIV (PLHIV) knowing their status in 2021 [a], similar to the proportion estimated 5 years ago [2].

The diagnosis gap is a known driver of the HIV epidemic [3]. The low uptake of HIV testing among cis-MSM and TGW has been attributed to meager options for testing in the Philippines [4, 5], limited currently to facility-based and community-based testing. The former is the more prevalent model [6] and involves using rapid diagnostic test (RDT) kits, available only in Department of Health (DOH)-accredited stand-alone laboratories, hospitals, and clinics, and is only facilitated by medical technologists specifically trained for HIV [7]. Whereas community-based testing is carried out by trained lay providers during community visits and outreach programs using RDT kits. To address the low uptake amid the limited choices, expanding options may be key to upscaling access and uptake of HIV testing. The World Health Organization (WHO) has recommended HIV self-testing (HIVST), which involves the use of RDT kits for individuals to perform and interpret on their own [8]. This may remove barriers in the current HIV testing in the Philippines, including geographical distance, lack of confidentiality or privacy, conflicting schedules, and stigma [4, 5, 9–12]. HIVST has been shown to be safe and effective at increasing uptake and frequency of HIV testing without compromising condom use, social safety, and enrollment to treatment [13]. In the Philippines, limited evidence shows acceptability and preference of blood-based over fluid-based tests among cis-MSM and TGW [14, 15].

An equally important approach is the use of technology-based interventions—this is the concurrent use of technology to expand reach, accelerate scale-up, and facilitate cost-efficient and instantaneous service delivery, responding to the inherent restrictions in face-to-face services [16]. Examples of these are online-based interventions, which if used with HIVST seem to synergistically remove barriers to HIV testing among cis-MSM especially among first-time testers [17, 18]. Even though the proportion of Filipinos accessing the internet (67.0%) and using social media (80.7%) are higher than the global average [19], this approach has not been maximized, yet has been increasingly used during the COVID-19 pandemic [20].

We aimed to describe the outcomes of a community-led online-based HIVST demonstration project done in Western Visayas, Philippines, particularly, in terms of reach, reporting and reactivity rates, and successful linkage to services. Furthermore, we aimed to explore the demographic, sexuality-, and context-related factors associated with HIVST-related behavior and preferences, particularly opting for DAH and willingness to share HIVST kits to their partners and peers.

## Methods

### Study design, setting, and participants

We did a multiple-center, retrospective cohort analysis of participants recruited in a community-led online-based HIVST demonstration in Western Visayas, Philippines, implemented from March to November 2020. The STROBE statement checklist of items was used to guide the development of this research [21].

Western Visayas is in the center of the Philippines and is composed of six provinces separated in three different islands. Its two highly urbanized cities (Bacolod City and Iloilo City) are HIV high burden areas [1]. HIVST was demonstrated in the region in 2020 by the DOH Western Visayas because, firstly, two thirds of new HIV cases in the Philippines are detected outside Metro Manila and the Western Visayas is among the areas with highest HIV incidence, contributing 6.2% of newly diagnosed cases in 2019 nationally [22], and secondly, almost one fourth of PLHIV in the region has not been diagnosed in 2019 [22].

The demonstration project was implemented by different CBOs led by the main study site, Family Planning Organization of the Philippines-Iloilo (FPOP-Iloilo)-Rajah Community Center. Both online and offline recruitment campaigns were conducted, using social media platforms and face-to-face invites in social and sexual networks, respectively. As the HIVST demonstration was only limited among individuals within the Western Visayas region, the campaigns were targeted among cis-MSM and TGW in the said region. These campaigns led interested individuals to an online sign-up sheet. All adults residing in the six provinces in Western Visayas who signed-up were eligible to receive the HIVST services from implementing CBOs which started to distribute INSTI® HIV Self-Test kits (BioLytical Laboratories, Richmond, British Columbia, Canada) in March 2020. While extreme lockdowns were implemented due to the unprecedented COVID-19 pandemic, the online-based nature of the program allowed continuity of provision of HIVST.

Using convenience sampling, we performed a secondary data analysis among people who opted and consented for the HIVST demonstration who fit the following inclusion criteria as follows: (1) self-identified as cis-MSM or TGW, (2) 18 years old and above, and (3) opted for online-based services. The following were excluded: (1) assigned female at birth, (2) assigned male at birth and identified as heterosexual, (3) opted for offline services, and (4) those who eventually disclosed that they were known PLHIV.

### Procedures

When signing up online, the participants were provided with pre-test and programmatic information. Upon providing their electronically recorded consent, data on demographics, sexual risk and behavior, and HIV testing related behavior and preference were collected through self-reporting. Thereafter, the participants were reached by the implementers through phone calls to verify the intent and data they provided. Participants accessed the HIVST package, either through pick-up or courier, containing the HIVST kit itself, instructional materials (containing information on HIV, on how to use, interpret, and dispose of the kit, on accessing the support hotline, and linkage to appropriate HIV-related services), and condoms and lubricants. Participants were followed-up through phone calls within two days upon access to determine the outcomes, to provide post-test counseling and support on linking them to appropriate services. For validation purposes, the participants were asked to show the outcome of the HIVST kit by sending a photo or through a video call. In rare cases when the result was invalid (*n* = 4), they were offered retesting using their preferred strategy (DAH or unassisted) but with a different HIVST kit.

Those who tested reactive were referred directly to HIV treatment facilities and follow-up calls were conducted at two weeks, four weeks, and then every four weeks until ART initiation or twelve weeks, whichever came first, to determine self-reported linkage to the cascade of HIV care services. Those who neither responded to follow-ups nor reported their cascade outcomes within twelve weeks were tagged as lost to follow-up. Verification of the self-reported cascade outcomes were legislatively possible only if they were eventually enrolled in the main study site, FPOP-Iloilo. Meanwhile, those who tested non-reactive were routinely provided with risk reduction counseling and were offered to be enrolled in the HIV preexposure prophylaxis (PrEP) program of FPOP-Iloilo (the only provider in the region during the span of the study) as part of the post-test counseling. The project officers in each CBO were designated to collect all the data using a standardized data collection sheet.

We created a research dataset for the purposes of the secondary analysis from the deidentified dataset from the implementers, which included participants who fit the study inclusion and exclusion criteria. We cleaned the dataset and ensured that recoding would preserve the original data as much as possible. The following outcomes were included: (1) HIVST result (reactive or non-reactive), (2) whether they opted for directly assisted (DAH) (i.e., in-person demonstration and / or supervision by a provider) or unassisted HIVST [8], (3) whether they were willing to distribute the kits to their partners or peers (i.e., secondary distribution of HIVST kits) or not [8], (4) linkage to appropriate HIV services, i.e., enrollment to care (confirmatory testing and treatment) among those reactive and prevention services (risk reduction counseling, condoms and lubricants, and/or PrEP) among those non-reactive, and (5) reports of adverse events such as suicidal attempts, coercion, and social harm [8, 23, 24]. Included covariates were (1) demographics (age, gender identity, and employment), (2) sexuality-related variables including (a) anal sex within the past 3 months, (b) number of male partners for the past 12 months, (c) history of HIV testing, i.e., first-time tester or not [25], and (d) source of information regarding the HIVST program, and (3) context-related variables such as (a) time, measured in the date of access of the HIVST service, and (b) place of residence. These variables were determined a priori [25, 26]. Some quantitative variables were transformed into categories, particularly, (1) age, grouped into less than or equal to 24 or 25 and over, signifying the young KP group [8], (2) number of male sexual partners in the past 12 months, grouped based on the median number based on national biobehavioral surveillance [4]. Some qualitative variables were recoded: (1) the extent of quarantine restrictions into “None to minimal” or “Maximal”, based on the date and location of the individual participation, and (2) the place of residence classified into either urban or rural.

### Statistical analysis

Descriptive statistics were done to summarize the predictors. We performed Chi-square and Fisher exact tests to compare baseline characteristics, stratified by reported HIV test result. To describe the outcomes of the HIVST demonstration, we determined the prevalence at each component of the testing cascade. Moreover, we performed multivariate logistic regression using complete case analyses and backward elimination to determine predictors associated with our outcomes of interest: (1) opting for DAH and (2) willingness to distribute. Predictors found to be statistically associated in the initial bivariate analyses using *p* < 0.25 were included in the final multivariate analyses. Chi-square tests were used to assess collinearity of potential predictors. We used c statistics and Hosmer-Lemeshow statistics to assess predictive power and model fit. We used *p* < 0.05 to determine significant outputs in Chi-square and Fisher exact tests and crude (cOR) and adjusted odds ratio (aOR). All analyses were performed using R version 4.0.3.

### Ethical approval

The study adhered to the principles of the Declaration of Helsinki and the ethical approval (NEC Code: 2021–004) was provided by the National Ethics Commission of the Philippine Council on Health Research and Development, Department of Science and Technology, Republic of the Philippines.

## Results

**Fig. 1:**
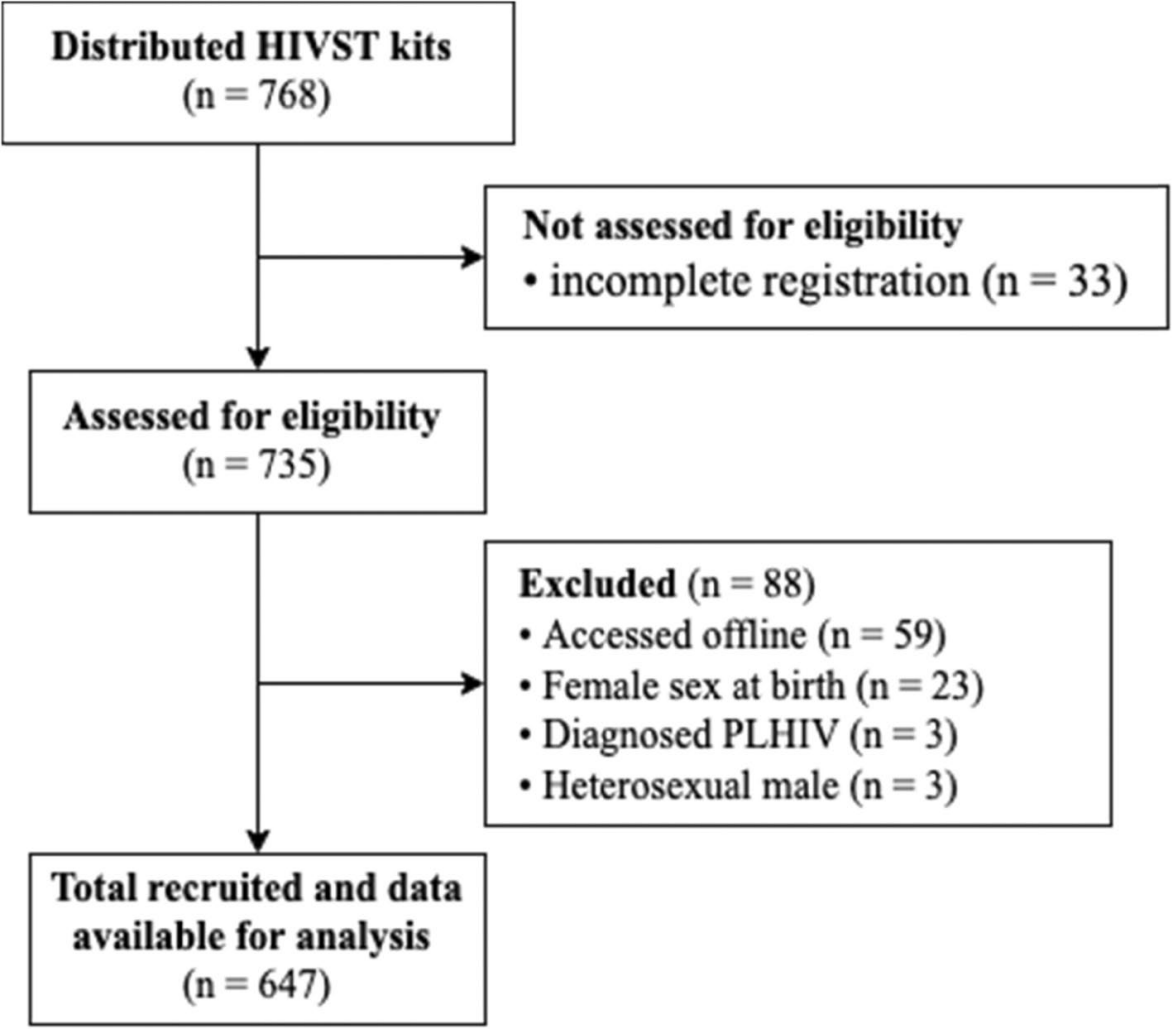
Flow diagram of the retrospective cohort study. HIVST – HIV self-testing, PLHIV – people living with HIV

From March to November 2020, 768 HIVST kits were distributed (Fig. 1). Due to missing documentation, 33 participants were not assessed for eligibility. Among those assessed, 88 were excluded based on the inclusion and exclusion criteria. Eventually, 647 participants were included in the analysis.

Median age of the participants was 26 (interquartile range 23–30) years. Majority self-identified as cis-MSM (91.2%), and most were employed (77.6%) and residing in an urban area (69.1%) (Table 1). Many (70.2%) had three or more sexual partners in the past 12 months and 51.8% had anal sexual intercourse in the past 3 months.

**Table 1:** Sociodemographic factors, sexual risk and behavior, and HIV testing-related behavior and preferences of the HIVST demonstration project participants, disaggregated into reported HIV testing result

	Distributed		Reported results (***N*** = 643)				
			Non-Reactive		Reactive		***p***-value
	n	(% of total of 647)	n	(%)^a^	n	(%)^a^	
**Age group**							0.441
18–24	243	(37.6%)	227	(93.4%)	16	(6.6%)	
25 and over	404	(62.4%)	367	(91.8%)	33	(8.3%)	
Missing	0	(0.0%)					
**Gender identity**							0.234^b^
Cis-MSM	590	(91.2%)	539	(92.0%)	47	(8.0%)	
Transgender woman	57	(8.8%)	55	(96.5%)	2	(3.5%)	
Missing	0	(0.0%)					
**Employment**							0.023 *
Unemployed	143	(22.1%)	139	(97.2%)	4	(2.8%)	
Employed	502	(77.6%)	455	(91.2%)	44	(8.8%)	
Missing	2	(0.3%)					
**Location**							0.556
Urban	447	(69.1%)	182	(91.5%)	17	(8.5%)	
Rural	200	(30.9%)	412	(92.8%)	32	(7.2%)	
Missing	0	(0.0%)					
**Number of male sex partners in the past 12 months**							0.757
Less than 3	190	(29.4%)	177	(93.2%)	13	(6.8%)	
3 or more	454	(70.2%)	417	(92.5%)	34	(7.5%)	
Missing	3	(0.5%)					
**Anal course in the past 3 months**							0.018 *
No	309	(47.8%)	293	(95.1%)	15	(4.9%)	
Yes	335	(51.8%)	300	(90.1%)	33	(9.9%)	
Missing	0	(0.5%)					
**First-time tester**							0.743
No	294	(45.4%)	269	(92.8%)	21	(7.2%)	
Yes	353	(54.6%)	325	(92.1%)	28	(7.9%)	
Missing	0	(0.0%)					
**Preference for assistance**							< 0.00 *
Unassisted	580	(89.6%)	539	(93.6%)	37	(6.4%)	
Directly-assisted	67	(10.4%)	55	(82.1%)	12	(17.9%)	
Missing	0	(0.0%)					
**Willingness to distribute HIVST kits to sexual and social network**							< 0.00 *
No	348	(53.8%)	308	(88.5%)	40	(11.5%)	
Yes	298	(46.1%)	285	(96.9%)	9	(3.1%)	
Missing	1	(0.2%)					
**Source of information about HIVST**							0.518^b^
Social network	616	(95.2%)	567	(92.6%)	45	(7.4%)	
Partner notification	24	(3.7%)	21	(87.5%)	3	(12.5%)	
Provider-initiated	7	(1.1%)	6	(85.7%)	1	(14.3%)	
Missing	0	(0.0%)					
**Community quarantine restrictions**							0.017 *
None to minimal	574	(88.7%)	526	(92.0%)	46	(8.0%)	
Maximum	73	(11.3%)	68	(93.2%)	3	(4.1%)	
Missing	0	(0.0%)					

^a^Denominator is number of individuals reported HIVST result disaggregated based on baseline characteristic

^b^Fisher exact test. All other comparison of proportions were done using Chi-square test

* significant at p < 0.05

*HIVST* HIV self-testing

Among those distributed with HIVST kits, more than half (54.6%) had never tested previously for HIV, most (89.6%) preferred unassisted HIVST, and almost half (46.1%) were willing to distribute kits to their sexual partners and peers. Furthermore, reporting rate of HIVST result was high at 99.3%.

Of the 643 who reported their HIVST outcomes, 49 (7.6%) tested reactive. The proportions of testing reactive were significantly higher among those employed (*p* = 0.023), who had anal intercourse in the past 3 months (*p* = 0.021), who opted for DAH (*p* = 0.018), not willing to distribute the HIVST kits (*p* < 0.000), and who accessed HIVST during none to minimal quarantine restrictions (*p* = 0.017) compared to their corresponding counterparts. There was no significant difference in the proportion of those tested reactive between first-time testers and those with a history of HIV testing (*p* = 0.743). Moreover, among those who tested reactive, 42 (85.7%) were eventually linked to care and 25 (51.0%) were initiated on ART during the study period (Table 2). Among those non-reactive, all 594 participants (100%) were provided prevention services through routine provision of risk reduction counseling and condoms and lubricants. Only 2 (0.3%) were successfully linked to PrEP services. Lastly, there were no reports of adverse events in the program.

**Table 2:** HIV self-testing demonstration study outcomes

	n / N (%)
**Distributed**	647
**Reported results**	643 / 647 (99.3%)
**Reactive**	49 / 643 (7.6%)
Linked to care^a^	42 / 49 (85.7%)
Initiated on antiretroviral therapy	25 / 49 (51.0%)
Lost to follow-up	7 / 49 (14.3%)
**Non-reactive**	594 / 643 (92.4%)
Linked to prevention services^b^	594 / 594 (100%)
Initiated on pre-exposure prophylaxis	2 /594 (0.3%)
**Adverse events reports**	0 / 647 (0.0%)

^a^Defined as being enrolled into a treatment facility

^b^Includes condoms and lubricants and behavioral risk reduction counseling

Only a few (10.4%) opted for DAH (Table 3). The likelihood of opting for DAH was higher among those who had three or more partners in the past year (aOR = 2.01 [CI = 1.01–4.35], *p* = 0.049) and among those who accessed HIVST during maximal quarantine restrictions (aOR = 4.25 [CI = 2.46–7.43], *p* < 0.00).

Almost half (46.1%) were willing to distribute the HIVST kits to their partners and peers (Table 4). The likelihood of willingness to share was higher among those residing in urban (aOR = 1.64 [CI = 1.15–2.36], *p* = 0.007), whereas it was lower among first-time testers (aOR = 0.45 [CI = 0.32–0.62], p < 0.00).

**Table 3:** Predictors of opting directly assisted HIVST

	Directly assisted HIVST				Crude OR		p-value	Adjusted OR		p-value
	n	/	N (% among distributed)		OR	(95% CI)		aOR	(95% CI)	
**Age group**										
18–24	21	/	243	(8.6%)	1.00					
25 and over	46	/	404	(11.4%)	1.36	(0.80–2.38)	0.269			
Missing	0	/	647	(0.0%)						
**Gender identity**										
Cis-MSM	64	/	590	(10.8%)	1.00			1.00		
Transgender woman	3	/	57	(5.3%)	0.46	(0.11–1.29)	0.197*	0.69	(0.16–2.08)	0.562
Missing	0	/	647	(0.0%)						
**Employment**										
Unemployed	11	/	143	(7.7%)	1.00					
Employed	55	/	502	(11.0%)	1.48	(0.78–3.05)	0.258			
Missing	2	/	647	(0.3%)						
**Location**										
Rural	42	/	447	(9.4%)	1.00			–		
Urban	25	/	200	(12.5%)	0.73	(0.43–1.24)	0.233*	–	–	–
Missing	0	/	647	(0.0%)						
**Number of male sex partners in the past 12 months**										
Less than 3	10	/	190	(5.3%)	1.00			1.00		
3 or more	56	/	454	(12.3%)	2.53	(1.32–5.38)	0.009*	2.01	(1.01–4.35)	0.049**
Missing	3	/	647	(0.5%)						
**First-time tester**										
No	40	/	294	(13.6%)	1.00			–		
Yes	27	/	353	(7.6%)	0.53	(0.31–0.88)	0.015*	–	–	–
Missing	0	/	647	(0.0%)						
**Source of information about HIVST**										
Social network	61	/	616	(9.9%)	1.00					
Partner notification	5	/	24	(20.8%)	2.39	(0.77–6.20)	0.093			
Provider-initiated	1	/	7	(14.3%)	1.52	(0.09–9.08)	0.702			
Missing	0	/	647	(0.0%)						
**Community quarantine restrictions**										
None to minimal	115	/	580	(19.8%)	1.00			1.00		
Maximum	36	/	67	(53.7%)	4.70	(2.79–7.95)	< 0.00*	4.25	(2.46–7.43)	< 0.00**
Missing	0	/	647	(0.0%)						

c statistic = 0.70; Hosmer-Lemeshow χ2 = 0.17433, df = 8, p-value = 1

* significant at < 0.25 for crude odds ratio (cOR); ** significant at < 0.05 for adjusted odds ratio (aOR)

**Table 4:** Predictors of willingness to distribute HIVST to sexual partners and peers

	Willingness to distribute				Crude OR		p-value	Adjusted OR		p-value
	n	/	N (% among distributed)		OR	(95% CI)		aOR	(95% CI)	
**Age group**										
18–24	100	/	242	(41.3%)	1.00			–		
25 and over	198	/	404	(49.0%)	1.36	(0.99–1.89)	0.058*	–	–	–
Missing	1	/	647	(0.2%)						
**Gender identity**										
Cis-MSM	280	/	589	(47.5%)	1.00			–		
Transgender woman	18	/	57	(31.6%)	0.51	(0.28–0.90)	0.023*	–	–	–
Missing	1	/	647	(0.2%)						
**Employment**										
Unemployed	61	/	143	(42.7%)	1.00					
Employed	236	/	501	(47.1%)	1.20	(0.82–1.75)	0.347			
Missing	2	/	647	(0.3%)						
**Location**										
Rural	223	/	447	(49.9%)	1.00			1.00		
Urban	75	/	199	(37.7%)	1.65	(1.17–2.32)	0.004*	1.64	(1.15–2.36)	0.007**
Missing	1	/	647	(0.2%)						
**Number of male sex partners in the past 12 months**										
Less than 3	83	/	189	(43.9%)	1.00					
3 or more	214	/	454	(47.1%)	1.14	(0.81–1.60)	0.456			
Missing	4	/	647	(0.6%)						
**First-time tester**										
No	172	/	294	(58.5%)	1.00			1.00		
Yes	126	/	352	(35.8%)	0.40	(0.29–0.54)	< 0.00*	0.45	(0.32–0.62)	< 0.00**
Missing	1	/	647	(0.2%)						
**Source of information about HIVST**										
Social network	281	/	616	(45.6%)	1.00					
Partner notification	13	/	23	(56.5%)	1.55	(0.67–3.68)	0.306			
Provider-initiated	4	/	7	(57.1%)	1.59	(0.35–8.13)	0.546			
Missing	1	/	647	(0.2%)						
**Community quarantine restrictions**										
None to minimal	43	/	348	(12.4%)	1.00			1.00		
Maximum	108	/	298	(36.2%)	4.03	(2.73–6.05)	< 0.00*	3.60	(2.41–5.45)	< 0.00**
Missing	1	/	647	(0.2%)						

c statistic = 0.70; Hosmer-Lemeshow χ2 = 1.1911, df = 8, p-value = 0.9967

* significant at < 0.25 for crude odds ratio (cOR); ** significant at < 0.05 for adjusted odds ratio (aOR)

## Discussion

We found that online-based HIVST reached many first-time testers among cis-MSM and TGW, similar with previous studies [27–31]. Reporting and linkage to care and prevention rates were high but ART and PrEP initiation were sub-optimal. Reactivity rate and HIVST preferences were associated with participants’ vulnerabilities and context.

It is striking that there seemed to be no difference in reactivity rate between first-time and ever testers, especially considering that in the Philippines all of those who come for HIV testing are routinely provided with risk reduction counselling [7, 32] which would be expected to decrease their risk for HIV. Our finding suggests that the aforementioned may have had marginal impact, as noted in other studies [33]. Nonetheless, HIV testing is a good avenue to educate KP regarding HIV and their risks. Hence, the DOH should not only consider reviewing its risk reduction counseling strategy but also advocate for and upscale all aspects of combination prevention [34], particularly pre- and post-exposure prophylaxis, condom use, and addressing stigma and discrimination, which have been determined as national priority interventions for HIV prevention [35].

Although only a minority in our cohort (10.4%) opted for DAH, the following findings have important implications for policy. Firstly, reactivity among those who opted for DAH was significantly higher compared to unassisted, similar with another study [36]. Moreover, we found that those with three or more sexual partners in the past year had twice higher odds of opting for DAH. There is evidence on the presence of anxiety related to the HIVST process, particularly linkage to care, and this translates to a desire for assistance among cis-MSM [14], TGW [14, 31], and other KP [37, 38]. Secondly, while it may be intuitive that testing for the first-time is associated with higher odds of DAH as seen in previous studies [14, 25, 39], it was the opposite in our bivariate model. Participants may have been enticed by the privacy, convenience, and independence that HIVST offers. Lastly, we found that stricter COVID-19-related quarantine restrictions were associated with higher likelihood of DAH. We could only speculate that the perceived limited access to healthcare services amid a time of public health crisis and uncertainty may have reinforced dependence on health providers and peers especially given that PLHIV and KP are at increased risk of vulnerability to both HIV and COVID and its physical, mental, and social comorbidities [40, 41]. Therefore, as DAH was associated with better retention [42] and higher ART initiation [43], even during the COVID-19 pandemic [44], implementation of HIVST in the Philippines should provide and expand options for direct assistance that go beyond in-person demonstration and also include emotional support [45]. This could involve capacitating community-based testing providers and “seeds” to provide demonstrations and peer support to their communities and networks [46, 47], respectively, and kits being delivered by trained providers themselves. Moreover, ensuring DAH may be crucial if the Philippines introduces oral fluid-based test, to address lack familiarity as Filipino KPs are more accustomed to blood-based tests.

Secondary distribution has been shown to increase the reach, positivity yield, and cost-efficiency of HIV testing among cis-MSM [26, 46, 48]. Like other studies which showed increased distribution [26, 49], we found that willingness to distribute was higher among those with prior HIV testing. This is reassuring as we also found that online-based HIVST can effectively reach to first-time testers, consistent with other studies [17, 18]. Hence, in the Philippines, where less than half (43%) of cis-MSM and TGW were ever tested for HIV [4], technology-based HIVST has the potential to increase the proportion of ever tested for HIV [17, 18] and, consequently, facilitate initial and repeat testing among their networks though secondary distribution [30, 50]. We also found that residing in urban areas was associated with increased odds of willingness to distribute. This may be due to the dense clustering of KP [51], higher access to queer culture [52] and HIV education [53], and higher acceptability of HIV interventions [51]. This is opportune as urban areas are priority sites for sustainable and effective HIV response [54]; as willingness was high, secondary distribution of HIVST kits could augment current HIV testing practices through approaches like index testing and sexual and social network testing [46, 47]. There is plenty of evidence that secondary distribution [55] and technology-assisted models [18, 55] play a role in increasing testing uptake among cis-MSM and TGW, whereas community-based models were found to be more effective among young people and male partners of females in antenatal clinics [55]. The knowledge gap on effective distribution models among other KPs, like PWID, people in prisons, and female sex workers, may be attributed to the disproportionately limited studies among these vulnerable groups. Hence, further studies are required to fully respond to their values and preferences on HIV testing.

Despite high rates of uptake, reporting, and referral to services, we found suboptimal initiation of antiretroviral interventions. Apart from the limitations brought by COVID-19, suboptimal initiation may be explained by the fact that only one in eleven CBOs in the demonstration was capable of prescribing ART or PrEP, similar to the experience in Thailand [43]. However, when treatment was also CBO-led, as in the HIVST demonstration in Vietnam, higher initiation rates was noted [56]. Furthermore, despite that rapid ART initiation has been recommended by the WHO since 2017, the current HIV treatment guidelines in the Philippines in 2018 did not mention this [b] and may explain the low ART initiation rate. Meanwhile, poor PrEP initiation may be explained by cost [57], especially that, unlike ART, PrEP is neither state-sponsored nor covered by health insurance in the Philippines. Overall, the benefits of online-based HIVST could not be maximized without concurrent innovations in treatment and prevention. Although a few treatment and PrEP facilities are CBOs or have partner CBOs in the Philippines, continuing the endeavor by the DOH to further decentralize HIV-related services to CBOs should be prioritized. Likewise, technology-supported interventions or seamless online-to-offline transition during ART or PrEP prescribing, linkage to care, and retention, should be considered and further studied. Lastly, local treatment guidelines should be revised to allow rapid ART initiation.

The primary strength of this study was the technology-based delivery of the demonstration project; this allowed numerous and precise data points to be used to explore associations. Furthermore, to our knowledge, this is the first association study to consider the potential influence of quarantine restrictions on HIV service delivery in the Philippines. Meanwhile, it is important to acknowledge some study limitations. Firstly, as this study is a secondary data analysis, we were bound to the limitations of the primary data collection such as high potential for information bias, as much of the data was collected through self-reporting which is particularly vulnerable to social desirability bias. However, verification was done whenever possible. Moreover, likewise with a previous study [58], the willingness to distribute HIVST kits were collected at baseline and, hence, may be influenced by the uncertainty of their HIV status. Secondly, the online-based convenience sampling may have led to self-selection bias. Generalizing the findings of our study must be done with caution. Lastly, there are limitations of in the use of stepwise backward elimination. Although it prevents overfitting and allow different combinations of variables [59–62], there is considerable variance when different samples are used [62] and there is potential for inappropriate variables to be included in the model [59, 60, 62]. We did, however, ensure that there were sufficient events per variable [60, 63] and that we explored a priori predictors, respectively. Thus, we are confident that the models predict the outcomes within the context of the study.

## Conclusions

We have shown that a community-based online-based HIVST intervention is safe and has the potential to increase uptake of HIV testing and linkage to appropriate service among cis-MSM and TGW, yet initiation of ART and PrEP were low. The study emphasized the importance of providing different options for HIVST which suite their values and preferences of KP. Geographical, temporal, and sociocultural contexts are important considerations in ensuring differentiated services are provided.

## Data Availability

Due to ethical reasons, the dataset created and analyzed is not publicly available as it contains potentially sensitive information. For further inquiries, email may be sent to the corresponding author, Dr. Patrick C. Eustaquio via patrick@loveyourself.ph.

## Abbreviations

AIDS: Acquired immune deficiency syndrome
aOR: Adjusted odds ratio
ART: Antiretroviral therapy
CBO: Community-based organization
CI: Confidence interval
cis-MSM: Cisgender men who have sex with men
cOR: Crude odds ratio
COVID-19: Coronavirus disease in 2019
DAH: Directly-assisted HIV self-testing
DOH: Department of Health
FPOP: Family Planning Organization of the Philippines
HIV: Human immunodeficiency virus
HIVST: HIV self-testing
KP: Key population
PLHIV: People living with human immunodeficiency virus.
PrEP: Pre-exposure prophylaxis
STROBE: Strengthening the reporting of observational studies
TGW: Transgender women
UNAIDS: Joint United Nations Programme on HIV/AIDS
WHO: World Health Organization
